# Diagnostic genomic sequencing in critically ill children

**DOI:** 10.1515/medgen-2023-2015

**Published:** 2023-06-13

**Authors:** Bernd Auber, Gunnar Schmidt, Chen Du, Sandra von Hardenberg

**Affiliations:** Hannover Medical School Department of Human Genetics Hannover Germany; Hannover Medical School Department of Human Genetics Hannover Germany; Hannover Medical School Department of Human Genetics Hannover Germany; Hannover Medical School Department of Human Genetics Hannover Germany

**Keywords:** rapid whole genome sequencing, clinical utility, precision medicine, OPS 1-944.11, diagnostic odyssey

## Abstract

Rare genetic diseases are a major cause of severe illnesses and deaths in new-borns and infants. Disease manifestation in critically ill children may be atypical or incomplete, making a monogenetic disease difficult to diagnose clinically. Rapid exome or genome (“genomic”) sequencing in critically ill children demonstrated profound diagnostic and clinical value, and there is growing evidence that the faster a molecular diagnosis is established in such children, the more likely clinical management is influenced positively. An early molecular diagnosis enables treatment of critically ill children with precision medicine, has the potential to improve patient outcome and leads to healthcare cost savings. In this review, we outline the status quo of rapid genomic sequencing and possible future implications.

## Introduction

While treatment options of common critical diseases in children (such as in neonatal respiratory distress syndrome) has improved significantly in the past few decades, genetic disorders are now a major contributor to infant morbidity and mortality. Rare genetic disorders often manifest as severe multisystemic disorders and represent a significant cause of pediatric hospitalisation. One third of all deaths in the first year of life is attributable to genetic disorders (Dodge et al., 2011) (Stevenson & Carey, 2004; Yoon et al., 1997). In a recent study, this rate was even higher, with a monogenetic genetic disease identified as the cause in 41 % of infant deaths. Treatment options, that have the potential to improve the outcome, would have been available for 30 % of these diseases (Owen et al., 2023). Therefore, a timely identification of an underlying genetic defect in a critically ill child is expected to influence treatment and outcome favourably. Until recently, three major obstacles prevented the successful implementation of molecular genetic diagnostics in the clinical regimen of these children: Firstly, the diversity of rare genetic diseases is staggering – the rare disease Orphanet database lists more than 6.100 diseases, 72 % of them are of genetic origin and almost 70 % are exclusively of pediatric onset (Nguengang Wakap et al., 2020). Historically, a plethora of genetic tests like karyotyping, chromosomal microarray and single gene or gene panel sequencing had to be performed sequentially, guided by the clinician’s differential diagnosis. This time consuming and ineffective process has been nicknamed as “diagnostic odyssey” (Petrikin et al., 2015).

Secondly, with an average turnaround time of several weeks or even months, genetic testing using array CGH or sequencing technologies was rarely able to provide clinicians in neonatal or pediatric intensive care units (NICU/PICU) with a timely test result, that allowed to influence the clinical course.

Thirdly, rapid genomic sequencing is still costly. Despite this fact, many studies have demonstrated overall cost saving effects when rapid genomic sequencing is used as a first-tier test in critically ill children. In some health-care systems, rapid genomic testing is already implemented as routine diagnostics, but most countries lack adequate reimbursement schemes, including Germany.

Here, we discuss the characteristics of rapid genomic sequencing and review the evidence regarding diagnostic and clinical utility and cost-effectiveness of rapid genomic testing in critically ill children.

## Shortening the diagnostic odyssey by genomic sequencing

Thanks to advances in high-throughput sequencing, genetic testing can now be carried out using whole exome or genome sequencing (WES/WGS). These so called “genomic” sequencing tests cover the majority of possible causes for rare genetic disorders. Therefore, many differential diagnoses can be tested for simultaneously and an agnostic approach can lead to diagnoses clinicians did not suspect in first place. This type of testing is able to speed up the diagnosis of a monogenetic disease compared to the historically done single gene testing approach. Especially in critically ill children, faster genomic test results more often not only make an impact on patient care but also lead to healthcare cost savings (Sanford Kobayashi & Dimmock).

## Speeding up genomic sequencing

Even this genomic testing strategy normally requires several weeks or months to complete in routine diagnostics. While it took more than a decade to sequence the first human genome (Lander et al., 2001; Venter et al., 2001), Stephen F. Kingsmore’s group was able to sequence the genomes of two critically ill children and provide a molecular diagnosis in 50 hours only a decade later (Saunders et al., 2012). The minimum turnaround time (TAT) for rapid WGS (rWGS) in a diagnostic setting is 13.5 hours (Owen et al., 2022). The shortest reported duration from receipt of sample in the laboratory to a preliminary diagnosis is now 7 hours 18 minutes (Gorzynski et al., 2022). These very rapid TATs are only achievable for selected samples in laboratories with specialized workflows.

However, decreasing TAT in genomic (WES or WGS) sequencing comes with a higher price-tag. This is mainly due to the fact, that genomic testing is expensive and economic processing of samples requires testing in groups (batching) to reduce the cost per sample (Stark et al., 2018). A simple way to accelerate the diagnostic process is to prioritize samples without changing the workflow. This might already result in a significantly shortened TAT of 2–3 weeks, but to achieve very rapid TAT <5 d, it is normally necessary to reduce the batch size. Using sequencing flow cells and/or sequencers with lower output is associated with (much) higher sequencing costs (Stark & Ellard, 2021). When using an Illumina NovaSeq 6000 sequencer, the most widely used system for WGS today, 20–24 genomes can be sequenced with the largest “S4” flow cell (output: 2.700 Gigabases), which requires a runtime of 44 h (300 cycles) and has a price per Gigabase (GB) of approximately 5 €. With the “S1” flow cell, which has an output of 450 GB, a trio genome can be sequenced with a significantly reduced runtime of 25 h but with a more than doubled cost per GB (approximately 11 €/GB; only sequencing kit costs – further costs for liquid handling robotics, sequencer, maintenance and computing, software, data handling/interpretation/storage, and staff apply). The estimated overall cost of diagnostic rapid trio WGS, the most widely used rapid sequencing method today, is approximately US$ 7,400 (average of total costs from cases sequenced at Rady Children’s Institute for Genomic Medicine, San Diego, USA, includes the cost of rapid trio WGS and interpretation) (Sanford Kobayashi et al., 2021).

Using different sequencing library preparation methods or less sequencing cycles has the potential to even further reduce the time necessary for the wet lab and sequencing process, but might also impact data quality.

Specialized bioinformatics pipelines and cloud computing can also accelerate data processing. Trio WES/WGS, the sequencing of the proband’s and both parental samples, facilitates variant data interpretation and allows to call *de novo* or biallelic variants immediately.

Automated phenotype extraction from electronic health records (Clark et al., 2019) and automated variant interpretation and treatment recommendation systems (Owen et al., 2022) have the potential to reduce TAT and workload simultaneously.

**Fig. 1:**
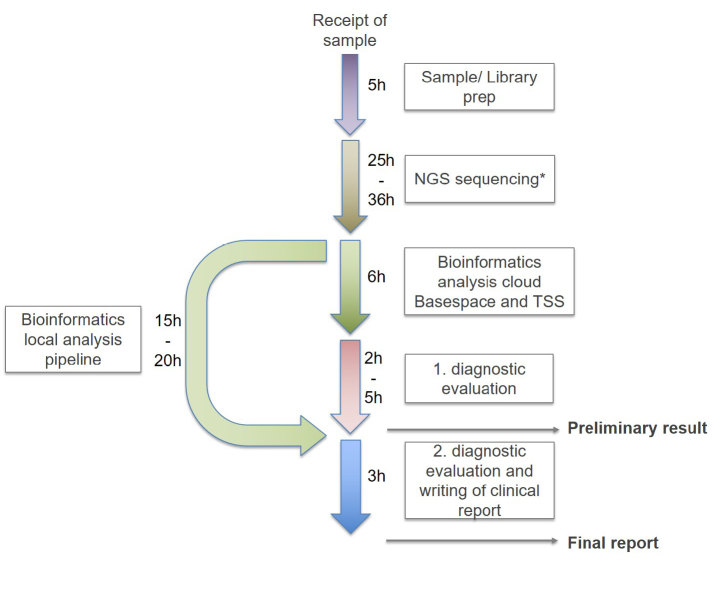
Timeline of rtWGS: Library preparation is performed directly from 10 µl of an EDTA blood sample without separate DNA extraction. 2x160 bp sequencing is performed on an Illumina NovaSeq 6000 instrument. Processing time including sequencing and bioinformatic analysis depends on the flow cell used (25 hours for a S1 flowcell and 36 hours for a S2 flowcell). Bioinformatic analysis is performed using both Illumina’s TSS cloud based and the locally installed GSVar sequencing pipelines. The two pipelines differ in the time required for bioinformatical analysis (TSS = 6h; GSVar =15-20h, depending on the performance of the local computing cluster). Manual variant interpretation typically requires two to five hours, accurate classification of variants using ACMG guidelines as well as drafting of a clinical report is estimated to be accomplished after 3 hours. A preliminary result can be communicated after 38–52 hours, a final written report after 50–69 hours.

To achieve rapid TAT, all steps of the workflow (from sample receipt to final report, see figure 1) might occur outside of normal working hours, increasing costs even further (Stark & Ellard, 2022). TAT of around two weeks (“rapid” genomic testing, rWES/WGS) are reported by most studies published to date. Only few larger studies (n=/>40) so far reported “ultra-rapid” genomic testing (urWES/WGS) with TATs <5 d (Dimmock et al., 2021; Gubbels et al., 2020; Lunke et al., 2020; Ouyang et al., 2021; Wang et al., 2020).

## Eligibility for rapid genomic testing

Given the extremely broad clinical spectrum of rare diseases in critically ill children, inclusion criteria for rapid genomic sequencing cannot be too specific without dramatically loosing sensitivity. Table 1 shows the testing criteria issued in 2019 by the first major health-care payer in California covering rapid genomic testing (Kingsmore & Cole, 2022), reflecting those used in published studies like NSIGHT2 or Project Baby Bear (Kingsmore et al., 2019) (Dimmock et al., 2021). Applying these broad and easy-to-use criteria in the Project Baby Bear study, the five participating tertiary care children’s hospitals had 4,300 admissions <1 year to NICU/PICU and 190 children (4 %) were determined eligible. 184 probands were included, resulting in rapid genomic sequencing with a high diagnostic yield.

**Tab. 1:** The July 2019 Blue Shield of California policy for coverage of rapid whole exome and whole genome sequencing in infants in intensive care units

Rapid whole exome sequencing or rapid whole genome sequencing, with trio testing when possible, meets the definition of medical necessity for the evaluation of critically ill infants in neonatal or pediatric intensive care with a suspected genetic disorder of unknown etiology when both (1 & 2) of the following criteria are met:		
1.	At least one of the following criteria is met:	
	a.	Multiple congenital anomalies (e.g., persistent seizures, abnormal ECG, hypotonia);
	b.	An abnormal laboratory test or clinical features suggests a genetic disease or complex metabolic phenotype (e.g., abnormal newborn screen, hyperammonemia, lactic acidosis not due to poor perfusion); or
	c.	An abnormal response to standard therapy for a major underlying condition.
2.	None of the following criteria apply regarding the reason for admission to intensive care:	
	a.	An infection with normal response to therapy;
	b.	Isolated prematurity;
	c.	Isolated unconjugated hyperbilirubinemia;
	d.	Hypoxic Ischemic Encephalopathy;
	e.	Confirmed genetic diagnosis explains illness;
	f.	Isolated Transient Neonatal Tachypnea;
	g.	Nonviable neonates.

## Diagnostic and clinical utility of rapid genomic testing

Over the past decade, starting with the successful clinical application of WES in a child with intractable bowel disease (Worthey et al., 2011), 31 clinical studies documented the diagnostic and clinical utility of genomic sequencing in critically ill children, comprising more than 2.800 tested neonates and infants (for a more detailed overview see (Kingsmore & Cole, 2022; Sanford Kobayashi & Dimmock; Stark & Ellard, 2022). Most of these studies have been observational, applying rapid or ultra-rapid genomic sequencing to a preselected patient cohort without a comparator. The reported average TAT differed widely, from 0.8 to 60 days (Clark et al., 2019; Scholz et al., 2021). Diagnostic yields varied between 19–83 %, depending on inclusion criteria and sample size, with an overall yield across all studies of 36 % (n=2.874). Kingsmore and Cole (2022) summarised clinical utility, measured by changes in clinical management after disclosure of test results in 29 studies. The mean rate of change in treatment (e. g. change in medication, surgery performed/not performed) was 27 % (7–63 %, n=2.222)(Kingsmore & Cole, 2022).

Three of these studies were performed as randomised controlled trials (RCTs). The first Newborn Sequencing in Genomic Medicine and Public Health (NSIGHT1) trial (Petrikin et al., 2018) randomised 65 NICU or PICU patients to receive rWGS plus standard genetic tests (*n* = 32, cases) or standard genetic tests alone (*n* = 33, controls). The study was terminated early due to loss of equipoise, with 73 % of controls receiving WGS as standard tests, and 15 % of controls undergoing compassionate cross-over to receive rWGS. Nevertheless, the rate of genetic diagnosis within 28 days of enrolment (the primary end-point) was higher in cases (31 %) than controls (3 %), and median time to diagnosis was significantly less in cases (13 days) than controls (107 days), establishing that rWGS increased the proportion of infants who received timely diagnoses of genetic conditions compared with standard genetic testing.

The second NSIGHT study was an RCT of the effectiveness of rWGS or rWES as first-tier tests in critically ill infants with diseases of unknown aetiology (Kingsmore et al., 2019). Of the 213 infants enrolled, 24 were very ill and were assigned to the urWGS group. The remaining infants were randomised, 95 to rWES and 94 to rWGS. The analytic performance of rWGS was superior to rWES but the diagnostic performance of rWGS and rWES were similar (19 and 20 %, respectively), as was time to result (both median 11 days). However, diagnostic yield of urWGS was higher than rWES/rWGS (46 %) and time to result was shorter (median 4.6 days).

Dimmock et al. (Dimmock et al., 2020) reported that clinicians involved in the NSIGHT2 trial perceived rWGS to be clinically useful in 77 % of infants. Both positive and negative tests were rated as having clinical utility (42 of 45 [93 %] and 112 of 156 [72 %], respectively). The clinical benefit in negative cases was explained by the reduced likelihood of a genetic condition, allowing physicians to focus on non-genetic aetiologies. Rapid WGS changed clinical management in 28 %, particularly in those infants receiving positive test results and those that were tested with urWGS. Outcomes of 32 (15 %) infants were perceived to be changed by rWGS. A third NSIGHT2 report evaluated parental perception (see respective chapter). The NSIGHT2 trial results established that rWES/WGS as a first-tier diagnostic test was considered advantageous by physicians and parents alike (Kingsmore & Cole, 2022).

In the third RCT, the NICUSeq Study Group randomized 354 infants aged <4 months in NICUs who had a suspected genetic disease to receive WGS either 15 or 60 days after enrolment (Group, 2021). At 60 days, 31 % of the infants in the early group received a molecular diagnosis and 21 % a change in management, compared with 15 and 10 % in the delayed group, respectively. The NICUSeq trial established that the introduction of WGS was associated with a significant increase in diagnostic yield and in focused clinical management compared with usual care (Kingsmore & Cole, 2022).

Measuring clinical utility was not standardized in the trials reported so far, making it difficult to compare the results of different studies. Most often, clinical utility is defined as the reported change in management from the referring clinician after disclosure of test results, which might be prone to bias. Measurement of clinical utility might become more standardized with the recent introduction of validated surveys like the Clinician-reported Genetic testing Utility InDEx (C-GUIDE) (Hayeems et al., 2022).

## Parental perception

When rapid genomic testing was introduced, there were concerns if parents were able to give informed consent for genomic testing while their child is critically unwell and that testing could cause anxiety and decisional regret due to the generation of complex, sometimes unexpected and life-altering information (Diamonstein, 2019).

A post-test survey issued to 117 families who participated in the NSIGHT2 RCT revealed that more than 90 % of parents felt adequately informed to consent to diagnostic genomic sequencing. 27 infants had received a genomic diagnosis, but 156 parents (97 %) reported, that testing was at least somewhat useful. Upon follow-up, harm was reported by 3 families (2 %). In 81 % (89) of 111 infants, families and clinicians agreed that genomic results were useful (Cakici et al., 2020).

As part of an Australian rWES implementation study (Lunke et al., 2020), surveys were sent to parents >12 weeks after genomic results return. 94 % of parents (n=50) reported receiving enough pretest information and 83 % (n=44) felt they had received adequate post-test counselling. Most parents reported no or only mild decision regret (n=45, 82 %) (Brett et al., 2020).

## Cost effectiveness

Despite the higher initial diagnostic costs of rapid genomic sequencing in critically ill children compared to standard of care, the overall cost efficacy of this approach has been demonstrated in several health care systems (e. g., in the USA, Australia, and Hong Kong) not only for neonatal but also for pediatric patients (Chung et al., 2020; Dimmock et al., 2021; Farnaes et al., 2018; Goranitis et al., 2022; Sanford Kobayashi & Dimmock): In a study of 42 infants that had received rWGS, overall average cost savings of US$19,000 per sequenced patient was achieved, resulting primarily from a reduction in hospital length of stay (Farnaes et al., 2018). Thereafter, the multisite “Project Baby Bear” collaboration (Dimmock et al., 2021) demonstrated that implementing rWGS as a first-line diagnostic test in 184 critically ill neonates resulted in a diagnostic yield of 40 % with a median TAT of 3 days. In 32 % of infants, rWGS testing led to changes in management These management changes resulted in additional costs of $9,492 cost for rWGS and precision medicine per child, but led to US$12,041-US$15,786 in cost savings per child sequenced (e. g. reduction of other diagnostic test procedures, reduced length of ICU stay). Post hoc sensitivity analysis showed that if the TAT was prolonged from an average of 3 days to 7 or 14 days, most of cost savings were lost due to increased inpatient days.

In Australia, another implementation project using rWES showed comparable cost-effectiveness: of 40 enrolled critically ill children, 21 received a diagnosis, resulting in a change of management in 12 of these patients. Cost per diagnosis was US$ 10,453, cost savings from avoidance of planned tests and procedures and reduced length of stay were estimated to be around US$ 10,600 per patient tested (Stark et al., 2018).

A study from Hong Kong reported that rapid trio WES in 102 families had a diagnostic yield of 31 % with a median TAT of 11 days. Cost analysis was performed in eight patients. Rapid WES was estimated to reduce hospital length of stay by 566 days for all eight patients combined and decreased healthcare costs by HKD $8,044,250 (US$ 1,036,503) for these eight patients. The net cost-savings after inclusion of rWES costs were estimated to be HKD$ 5,325,187 (US$ 686,151) (Chung et al., 2020).

In summary, data from different healthcare systems in several countries support the notion that rapid genomic sequencing does in fact reduce the cost of care.

## Implementation of rapid genomic sequencing in national health care systems

Because of the great benefits in the treatment of critically ill children reported in the studies reviewed above, rapid genome sequencing is becoming routine in an ever-increasing number of countries (Jezkova et al., 2022). The National Health Service (NHS) in England has implemented rWES for critically ill children since October 2019 (Williamson et al., 2021). The NHS in Wales is the first service in the United Kingdom to introduce a national diagnostic rWGS service for critically ill newborns and children as a front-line test. The “Wales Infants’ and childreN’s Genome Service” (WINGS) was launched in April 2020 (Murch et al., 2021)*.*

In the USA, based on the success of Project Baby Bear, the “Ending the Diagnostic Odyssey Act 2021” was introduced which allows all 50 state Medicaid programs to cover rWGS for eligible individuals. In September 2021, Michigan became the first US state to reimburse for rWGS in critically ill Medicaid-covered infants in NICUs and PICUs, priced at US$6,275 for single and US$10,750 for trio rWGS (Kingsmore & Cole, 2022).

Australia is implementing rapid genomic testing via the Australian Acute Care Genomics program in the Australian public health care system (Lunke et al., 2020).

In Germany, procedures for inpatients are reimbursed via an “operations and procedures key” (OPS) system. For “extensive molecular testing” in newborns and children below 14 years, a specific OPS code (“1-944.11”) can be used, but multiple criteria like such as extensive laboratory and radiologic testing must be fulfilled before this OPS code can be applied. Even in critically ill children, these criteria are not met in all cases and the reimbursement for this OPS code must be negotiated individually by each hospital. Using this OPS code, the diagnostic yield of WES was determined in 61 critically ill infants with an unknown underlying disease within the first year of life in University Medical Center Hamburg-Eppendorf. A definitive genetic diagnosis within that cohort was achieved in 28 children (46 %) (Scholz et al., 2021).

Since May 2022, the first German multicenter study on clinical utility of rWGS in critically ill children is recruiting (Baby Lion study, DRKS-ID DRKS00025163). Critically ill children in NICUs and PICUs across 12 German university and non-university hospitals are eligible for study inclusion. Pediatric patients (0–14 years) and preferably both parents are included based on consultations at interdisciplinary video conferences, where results are also discussed. Clinical utility is measured using the previously mentioned C-GUIDE survey and parental perception is monitored using a short second survey. Until now, a causative variant was reported in 45 % of patients (n=23) and highly likely causative variants of unknown significance were reported in another 6 % (n=3). Mean TAT from sample reception to disclosure of preliminary results was 2,9 days. Steps and minimum times for rapid genetic testing in this study are presented in Figure 1.

In 2024, the German national pilot genome sequencing project “Modellvorhaben Genomsequenzierung” will commence, and implementation of rWGS as a diagnostic test in critically ill children seems within reach. But at the moment it is unclear if rapid genomic sequencing in critically children will be funded (adequately) within this project.

## Conclusion

Technical advances in sequencing technology in recent years have made it possible to sequence and diagnostically evaluate a human genome within a few days at a reasonable cost. Applied to critically ill children, this has the potential to revolutionize the diagnostic work-upof these patients, enabling personalized treatment. The clinical usefulness of the genomic results has already been proven to be high, which has led to the implementation of rapid genomic sequencing as first-line diagnostic in some countries. To facilitate the broad adoption of rapid genomic sequencing, sufficient funding needs to be secured.

In the future, the addition of orthogonal methods such as transcriptome (Cummings et al., 2017) or long read sequencing (Gorzynski et al., 2022) may lead to a further increase in diagnostic yield.

A molecular diagnosis can trigger the development and application of individually designed, tailored therapies using patient-customized oligonucleotide molecules within a few months (Kim et al., 2019), offering possibilities for the treatment of rare and ultrarare genetic disease that were unthinkable a few years ago. In particular rapid genomic sequencing in critically ill infants offers a superior starting point for the timely initiation of such a therapy.

In Germany, too, the legal regulations will hopefully soon be in place to minimize the “diagnostic odyssey” by integrating medically indicated rapid genomic sequencing in critically ill children, even beyond studies.
